# How systemic racism results in poorer outcomes for First Nations, and what First Nations are doing about it: the example of kidney health

**DOI:** 10.1186/s12913-025-13179-6

**Published:** 2025-08-18

**Authors:** Josée G. Lavoie, Lorraine McLeod, James Zacharias, Tannyce Cook, Reid Whitlock

**Affiliations:** 1 First Nations Health and Social Secretariat of Manitoba, Winnipeg, Canada; 2 University of Manitoba, Winnipeg, Canada; 3 Seven Oaks Hospital, Winnipeg, Canada

**Keywords:** Indigenous, Primary care, Remote, Rural, Self-determination, Racism, Renal, Canada, Manitoba

## Abstract

**Background:**

End-stage kidney disease continues to disproportionally impact the lives of First Nations peoples. Systemic racism is a key determinant, and manifests as differential access to determinants of health (housing, employment, access to care) and differential care. This paper discusses how different models of primary healthcare operating in rural and remote Manitoba communities results in different outcomes for patients identified as being at risk of kidney disease.

**Methods:**

This study is a partnership between researchers from the First Nations Health and Social Secretariat of Manitoba and the University of Manitoba. We used health administrative data held at the Manitoba Centre for Health Policy for the period of 2006-2019, linked to the Manitoba First Nations Research File to identify First Nations. We compared rates of laboratory follow-up tests, nephrology consults, PHC visits, and hospitalizations between different models of care using a negative binomial regression model adjusted for age, sex, eGFR heat-map category, urine ACR heat-map category, and Elixhauser comorbidity index.

**Results:**

We identified 12,613 First Nations people with chronic kidney disease (CKD) during the study period. First Nations individuals with CKD who reside in communities served by Nursing Stations (most remote communities) when supplemented by additional Indigenous programs were consistently more likely to receive follow-up serum creatinine (OR 1.37, 95% CI: 1.30-1.45, *p*<0.001), urine ACR (OR 1.22, 95% CI: 1.16-1.28, *p*<0.001), serum potassium (OR 1.40, 95% CI: 1.32-1.49, *p*<0.001) than individuals who lived in communities served by Nursing Stations alone, Health Centres, Health Offices, or Off Reserve.

**Conclusions:**

Our results show that addressing the rise in premature mortality experienced by First Nations from kidney diseases require greater investments in First Nations-centric primary healthcare, that is locally managed. Additionally, off-reserve primary healthcare services must be alerted to their need to better address the needs of First Nations at risk of CKD, with more consistent follow up, referrals, and in providing culturally safe care. Finally, First Nations-led research in kidney health and primary healthcare is leading to significant improvements in outcomes, and needs to be better supported and resourced, and imbedded in a context of greater investments to improve access to all determinants of health and counter systemic racism.

## Introduction

While a diagnosis of end stage kidney disease (ESKD) is devastating for all, for Canadians living in rural and remote communities, this diagnosis may imply relocating to an urban or regional centre and increased distance from family and community at a time where emotional and logistical support is much needed. Relocation to an urban centre is usually required for the initiation of dialysis: in Manitoba, this primarily occurs in Winnipeg, the capital, and generally takes a few weeks. A return home may be possible if home modalities (home hemodialysis or peritoneal dialysis) are feasible and acceptable [1]. A return home or close to home may also be possible if institutional dialysis is the preferred option, provided that home is close to one of Manitoba’s 17 rural dialysis units, and space is available.

Financial hardship can be quite severe since relocation often results in loss of employment. For First Nation (FN) peoples, added negative experiences of the healthcare system, including repeated instances of anti-Indigenous interpersonal racism [2–4], embedded in and to some extent resulting from systemic racism [5, 6], can be important factors when deciding to seek primary healthcare (PHC, which includes out-patient PHC delivered by family physicians or nurse practitioners, as well as prevention-oriented services, public health and advocacy) support to maintain health or to initiate treatment [7]. Komenda and colleagues documented a 2-fold higher prevalence of CKD in Manitoba FN in comparison to the general population and a prevalence of severely increased albuminuria that was 5-fold higher [8].

Poverty and racism are independent factors for high rates of ESKD [9, 10], which interact to create disparities, especially in relation to accessing preventive care and treatment [11–13]. In Manitoba, Martens et al. [14, 15] showed that tribal councils with higher prevalence of diabetes, the single most common cause of ESKD in the FN population, had significantly lower average household income. While income might correlate with education, it also correlates with choices, providing FNs with opportunities to site-step problematic providers and travel longer distances to access better care. Across Canada, the disparities in FN kidney disease is understood as exacerbated by the interaction of remoteness, associated with infrastructure gaps, barriers to accessing primary health care, low socio-economic status ([14] SES, [16]), institutional racism (i.e. discriminatory explicit and implicit policies associated with specific institutions, such as a hospital) and the lingering yet continued effects of colonization [7]. Still, studies that disaggregate analyses on a per FN community basis have consistently shown differences across remote communities [17–22], suggesting that remoteness is not a decisive factor, and needs to be unpacked to truly comprehend determinants of better renal health outcomes for FNs.

This paper focuses on how different models of PHC perform in addressing the therapeutic needs by FN peoples living with or at risk of CKD and ESKD. While focusing on PHC, we wish to highlight how structural barriers to care, themselves a result of structural racism, have and continue to perpetuate worse outcomes among FN peoples at risk of kidney diseases. PHC can mitigate the impact of structural racism to some extent and can delay the onset of CKD and its progression to ESKD [23], however PHC alone cannot turn the tide, especially if narrowly defined as a time-limited episode of care between a provider and a patient (often called PHC). The First Nations Health and Social Secretariat of Manitoba (FNHSSM) has advocated for a broader and First Nation-defined concept of PHC [24–27] that more readily aligns with the World Health Organisation’s definition [28, 29]. In this paper, we highlight the impact of different models of PHC, and position these findings within the broader context of persisting structural racism.

## Background

Although multifactorial, systemic racism play an important role in the perpetuation of health inequities [30]. Systemic racism refers to the “economic, social and political institutions and processes of a society that create and reinforce racial discrimination” ([31], p. 5). Stelkia refers to these multiple and intersecting pathways as the“compounding weight” ([30], p. 6) of systemic racism. Examples are provided below. The list is by no means comprehensive. 

- Colonial legislation and policies perpetuating structural deprivation: for examples, the legacy of residential schools met with dismal investment in healing and mental health initiatives [32, 33]; under-investments in infrastructure leaving to continued housing pressures [34–36]; decades of compromised access to safe drinking water as a result of jurisdictional wrangling [37];
- Systems of failure, harm, and indifference: for example, Child Protective Services which focus on assessing the risk to children rather than the needs of families, resulting in the disproportionate FN loss of parental rights and trauma, that have become normalized [38];
- Impacts on access to healthcare: for examples, practices of denial, dismissal or of differential care associated with interpersonal racism [6, 30, 39] fostering fear and distrust; compounded by what seems to be a capricious system of approval to access medical transportation and other benefits guaranteed by policy [7]; and.
- Increased risk factors for chronic disease and poor health: at an individual level, socio-economic status can mitigate but cannot eliminate the compounding impact of factors already mentioned, to which may be added instances of food insecurity [40] impacting access to affordable nutritious food; and triggering coping strategies that might be counterproductive [41].

The Truth and Reconciliation Commission calls on

“governments to acknowledge that the current state of Aboriginal health in Canada is a direct result of previous Canadian government policies, including residential schools, and to recognize and implement the health-care rights of Aboriginal people as identified in international law, constitutional law, and under the Treaties” (Call 18, [42], p. 2).

In this article, we position FNs’ disproportionate rates of ESKD as consequences of these policies. While we focus on access to care, we recognize that alone, better PHC can potentially delay the development of chronic kidney disease (CKD) and its progression to ESKD: the prevention of CKD requires addressing systemic racism and its impact on access to all determinants of health [43, 44]. Until this occurs, PHC will continue to play a pivotal role in ensuring that FNs can access timely screening, preventative care and treatment, to delay the progression of CKD to ESKD. This article compares the performance of different models of PHC delivery, on the quality of care in responding to the therapeutic needs of FN individuals living with CKD.

### First Nations peoples’ access to care across Manitoba: understanding the context

Although not a solution to systemic racism, improving FN peoples’ access to quality, responsive preventative services and PHC is crucial to mitigate the impact of some of the factors discussed above. Across Canada, FN peoples access PHC from a variety of providers. Communities served by federally-funded Nursing Stations have access to some PHC delivered on-reserve by federally-employed nurses working with an expanded scope of practice. Although structural barriers can be complex (scarcity of providers, funding and jurisdictional barriers) [18], some communities have succeeded in supplementing this model of care with visiting family physicians (FPs). This model of care has been associated with lower rates of hospitalization for Ambulatory Care Sensitive Conditions [17, 19, 21, 22, 45, 46]. These communities are generally remote and most often fly-in communities.

For the past 40 years, 12 Manitoba FN communities served by Nursing Stations (22,010 FN individuals based on 2017 data) have received PHC delivered on-reserve by Family Physicians (FPs) and other professionals employed by Ongomiizwin Health Services (OHS), an Indigenous-centric University of Manitoba PHC organization (since 2008) led by a team of Indigenous and non-Indigenous health professionals committed to implementing trauma informed care. Other FN communities, those that are within 250 km of a town, can access PHC through FPs in private practice (fee-for-service practices) working in rural communities (e.g. The Pas, Flin Flon, Thompson, Dauphin, Brandon, Winnipeg, Portage La Prairie), if such a practice exists [47]. Access to quality, responsive PHC in rural and remote settings is however hampered by volume, attrition, vacancies, and a dependence on irregular availability of traveling FPs working on rotation [48]. Off-reserve health service delivery is part of the overall Manitoba health care system, which despite challenges, is regulated by policy and a strategic redistribution of resources to ensure coverage. In these communities, on-reserve health services are limited to prevention. Manitoba reports the largest FP shortage in Canada, disproportionally impacting rural and remote communities [49]. To address this issue, Manitoba created a locum program where physicians split their time between urban and rural/remote practice, providing relief to rural providers and serving underserved communities.

The First Nations Health and Social Secretariat of Manitoba’s (FNHSSM’s) Diabetes Integration Project (DIP) provides screening and follow up of FN individuals. DIP-affiliated providers regularly screen for risks associated with kidney diseases and provide referral. The DIP utilizes Mobile Diabetes Health Care Service Delivery Teams to provide diabetes care and treatment services in 18 FN communities throughout Manitoba. Each team is comprised of two (2) nurses serving an overall population of approximately 8,000 people. At present, the DIP operates in addition to and in partnership with community-based services funded by the federal First Nation and Inuit Health Branch of Health Canada (FNIHB) [50, 51]. The DIP’s mobile teams systematically screen for kidney function, and refer FN individuals considered at low or moderate risk of CKD to PHC; those at high risk of CKD are referred to nephrologists. The program, which has been operating since 2008 in 19 of Manitoba 63 FN communities because of financial constraints, reports positive outcomes [52]. In addition to the DIP, and in an attempt to improve system’s responsiveness and address systemic racism, FNHSSM is leading the implementation of a number of initiatives, including:

- Anti-Racism Training for Health Care Providers: FNHSSM delivers an 8 weeks virtual program, available to selected provider groups;
- Virtual Kidney Check - Create a public health screening program using an integrated health dataset and tele nephrology for screening, risk stratification and management of chronic kidney disease (CKD) in First Nations adults across Manitoba [53].
- Kidney Check - This project is bringing kidney, diabetes and blood pressure checks to First Nations communities across western Canada [54].
- In Utero Exposure to Type 2 Diabetes and long-term risk of renal disease in offspring: Analysis of prospective cohort data from the iCARE Longitudinal follow-up study and the Manitoba Center for Health Policy Data (MCHP) Repository [55].
- First Nation co-designing local health services and advocacy (foot care, dialysis, retinal screening, access to family physicians, physician assistants and specialists).

Recent developments in Manitoba have raised expectations of PHC providers in the follow-up of FN patients with CKD. In a 2003 screening study of 468 FN individuals, 17% of those with albuminuria did not have diabetes or hypertension, suggesting that a broader etiology needs to be included in addressing kidney disease [56]. Current Clinical Practice Guidelines (CPG) created by the Manitoba Renal Program (MRP), recommend yearly screening for all at risk individuals, including all FN individuals over 10 years of age [57], based on a Risk-Based Triage put into place to triage referrals using a Kidney Failure Risk Equation Reference [58]. The intent was to eliminate virtually any wait time to see a nephrology team for patients at high risk of progression to ESKD. This approach identified all Manitoba FNs at high risk, thereby justifying yearly screening of all FNs over 10 years of age (estimated at 52,245 FN individuals in 2017). The extent to which these clinical guidelines are implemented by PHC providers (including on-reserve, OHS and other PHC providers) is not known. The yearly screening of 52,245 FN individuals raises issues of high sensitivity countered by a low specificity which may lead FPs to question this approach. Further, extending timely care to those deemed at risk may be beyond what the Manitoba healthcare system can deliver now, or before the COVID-19 pandemic, because of the scarcity of providers working in FN and rural/remote communities, high demand on their time, resulting in a demand-driven PHC system where immediate health concerns take precedence over effective chronic disease prevention. This is compounded by the discontinuities in PHC providers mentioned above. The FINISHED study demonstrated that targeted screening using point-of-care testing equipment in remote communities was highly-cost effective [59].

Evidence from the United States (1995–2010) shows that once the Indian Health Services adopted an integrated approach to the prevention and treatment of CKD, the incidence of ESKD began to decrease [60–65]. Translating this success to the Manitoba context will require working across jurisdictions and providers (Manitoba Health and the Reginal Health Authorities at the provincial level, OHS funded by the federal and provincial governments, and First Nation communities which are funded by the federal government for a limited complement of health services, supplemented by PHC nursing provided by the federal government), thus the need for an integrated strategy.

## Methods

This retrospective cohort study examines the performance of different models of PHC in meeting the treatment needs of FN individuals living with CKD.

### Partnership

The IK-Health Project was created as a partnership between the First Nation Health and Social Secretariat of Manitoba (FNHSSM), Manitoba Renal Program (MRP), and the University of Manitoba. The overall aim of the project is to improve FN peoples’ access to quality and responsive renal care across the continuum of health services, with a specific focus on PHC services. It was funded by the Canadian Institutes for Health Research (#387891).

### Ethics

This study received ethics approval from the University of Manitoba Health Research Ethics Board (File number H23086) and the Health Information Privacy Committee (HIPC). In addition, approvals were obtained from the Health Information Research Governance Committee (HIRGC). HIRGC, anchored within FNHSSM, holds the responsibility of authorizing/approving/reviewing ethics applications which include FN community members. Because this study used data from de-identified administrative databases that did not include names and addresses, a waiver to obtain individual informed consent was provided by the Health Information Privacy Committee (HIPC, recently renamed the Provincial Health Research Privacy Committee, or PHRPC), in accordance with the Manitoba Personal Health Information Act.

### Data sources

We used health administrative data held at the Manitoba Centre for Health Policy (MCHP) which was linked to the Manitoba First Nations Research File to identify FN. The Manitoba First Nations Research File is a registry of all people in Manitoba who qualify as Status Indians under the Indian Act and is maintained by the FNHSSM. Other databases included: Diagnostic Services Manitoba Laboratory Data, Medical Claims, Hospital Discharge Abstracts, Drug Program Information Network Data, Public Canadian Census Files, Manitoba Health Insurance Registry, and Physician Resource File. All records are de-identified and can be linked to unique individuals through a scrambled personal health identification number.

### Study population

All FN people in Manitoba over the age of 10 with an incident diagnosis of chronic kidney disease (CKD) between April 1, 2006 and December 31, 2019 were eligible for inclusion. An individual was identified as FN if they were recorded in the Manitoba First Nations Research File. We used a local case definition to ascertain diagnoses of CKD [58]. The case definition for CKD was either 2 abnormal estimated glomerular filtration rate (eGFR) or urine albumin to creatinine ratio (ACR) test results > 90 days apart in laboratory data, an ICD-9 or ICD-10 diagnosis code indicative of CKD in Medical Claims or Hospital Discharge Abstracts, or a prescription indicative of CKD in the Drug Program Information Network Data. We calculated the eGFR with the CKD-Epidemiology Collaboration study equation for adults and the Pediatric Schwartz formula for children, imputing 3rd percentile height [66, 67]. We converted urine protein to creatine ratio test results to urine ACR values using an equation developed by Weaver and colleagues [68]. The first date where one the conditions of the CKD case definition was met was selected as the index date. Individuals were excluded from the cohort if they had a diagnosis of CKD prior to April 1, 2006 or if they were on chronic dialysis, had a kidney transplant, or were ≤ 10 years of age, prior to the index date. Chronic dialysis was defined using an algorithm incorporating tariff codes and the Manitoba Renal Program’s kidney health records and kidney transplants were identified using tariff codes or hospital procedure codes [58].

### First Nations communities

There are 63 FN communities (reserves) in Manitoba. Each community’s health services are provided by a Nursing Station, a Health Centre, or a Health Office with supplementation provided by OHS and/or DIP. We used the Manitoba Health Insurance Registry to determine what address an individual in our cohort was residing in at the time of the index date and then cross-referenced with the communities to group the individuals into receiving health services from: Nursing Station alone, Nursing Station + DIP, Nursing Station + OHS, Nursing Station + OHS + DIP, Health Office alone, Health Office + DIP, Health Office + OHS, Health Centre alone, or Health Centre + DIP. Individuals residing off-reserve at the time of the index date in Manitoba’s 2 major urban centres (Winnipeg and Brandon) were grouped into Off-Reserve Urban, and individuals living in other communities in Manitoba off-reserve were grouped into Off-Reserve Rural.

### Outcomes

Our primary outcomes were occurrences of follow-up laboratory tests, nephrology consults, PHC visits, hospitalizations, and prescription drugs post-index date. For follow-up laboratory tests, we assessed serum creatinine, urine albumin to creatinine ratio, serum potassium, serum albumin, hemoglobin a1c (HgbA1c), and cholesterol testing done in an outpatient setting from the Diagnostic Services of Manitoba laboratory database. Nephrology consults were ascertained from the Medial Services database. PHC visits were defined as a visit to a FP or a nurse practitioner as documented in the Medical Services database. Hospitalizations were ascertained from Hospital Discharge Abstracts. We looked at inpatient hospitalizations overall as well as hospitalizations related to ambulatory care sensitive conditions (ACSC) [17, 58]. For our follow-up assessment of drug prescriptions, we assessed prescriptions of angiotensin-converting-enzyme (ACE) inhibitors, angiotensin II receptor blockers, statins, diuretics, β-blockers and calcium channel blockers using Anatomic Therapeutic Chemical codes from the Drug Prescription Information Network.

### Variables

We collected demographics, CKD severity, comorbidities and drug prescriptions for each individual at baseline. In addition to community of residence, we also collected age and sex from the Manitoba Health Insurance Registry. We classified patients by CKD severity according to Kidney Disease Improving Global Outcomes eGFR and urine ACR heat map with modifications to add classifications where one or both test results were missing at baseline [58, 69]. We used the most recent measurement on or prior to the index date. We collected all comorbidities in the Elixhauser Comorbidity Index using an algorithm by Quan and colleagues where we looked back at medical claims and hospitalizations from inception of the databases until the index date [70, 71].

### Statistical analysis

We reported baseline characteristics for the overall cohort and for each FN health service provider group. We reported categorical variables as a frequency and percentage and we reported continuous variables as a mean and standard deviation or as a median and interquartile range for variables that were not normally distributed.

Crude rates of laboratory follow-up tests, nephrology consults, PHC visits, and hospitalizations were calculated as number of events divided by person-time at risk and were presented as number of events per person-year. Observation time for each individual was censored at death, migration from the province, chronic dialysis, kidney transplant, or loss to follow-up. Time spent in a hospital stay post-admission was deducted from the person-time at risk. Adjusted rates of these events were calculated using a Poisson regression model adjusted for age, sex, eGFR heat-map category, urine ACR heat-map category, and Elixhauser comorbidity index.

We compared rates of laboratory follow-up tests, nephrology consults, PHC visits, and hospitalizations between FN health service provider groups using a negative binomial regression model adjusted for age, sex, eGFR heat-map category, urine ACR heat-map category, and Elixhauser comorbidity index. Results of all the comparisons were presented as odds ratios (OR) with 95% confidence intervals (CI). We also executed the same models where individuals residing in communities served by Nursing Stations + OHS or DIP were compared with all other communities.

We compared the proportions of prescriptions of angiotensin-converting-enzyme (ACE) inhibitors or angiotensin II receptor blockers, statins, or other anti-hypertensives (diuretics, β-blockers and calcium channel blockers) in the 18 months before the index date with the proportions 18 months after the index date using McNemar’s test. We made these comparisons in the overall cohort and within each health service provider group. All analyses were performed using SAS 9.4.

## Findings

We identified 12,613 FN people with an incident diagnosis of chronic kidney disease (CKD) during the study period. The mean age of the cohort was 50.5 ± 17.5 years with 562 (4.5%) below the age of 18. There were 7,159 (56.8%) who were female (Table 1).

**Table 1:** Baseline characteristics

*N*	12,613
Age (years)	50.5 ± 17.5
Age 11–17	562 (4.5%)
Age 18+	12,051 (95.5%)
Sex (Female)	7159 (56.8%)
Elixhauser Comorbidities Index	6.7 ± 3.3
Community of Residence	
Health Centre + DIP	600 (4.8%)
Health Centre Only	948 (7.5%)
Health Office + DIP	619 (4.9%)
Health Office + OHS	176 (1.4%)
Health Office Only	824 (6.5%)
Nursing Station + DIP	707 (5.6%)
Nursing Station + DIP + OHS	101 (0.8%)
Nursing Station + OHS	2161 (17.1%)
Nursing Station Only	1486 (11.8%)
Off Reserve - Rural Remote	1550 (12.3%)
Off Reserve - Urban	3441 (27.3%)

**Fig. 1:**
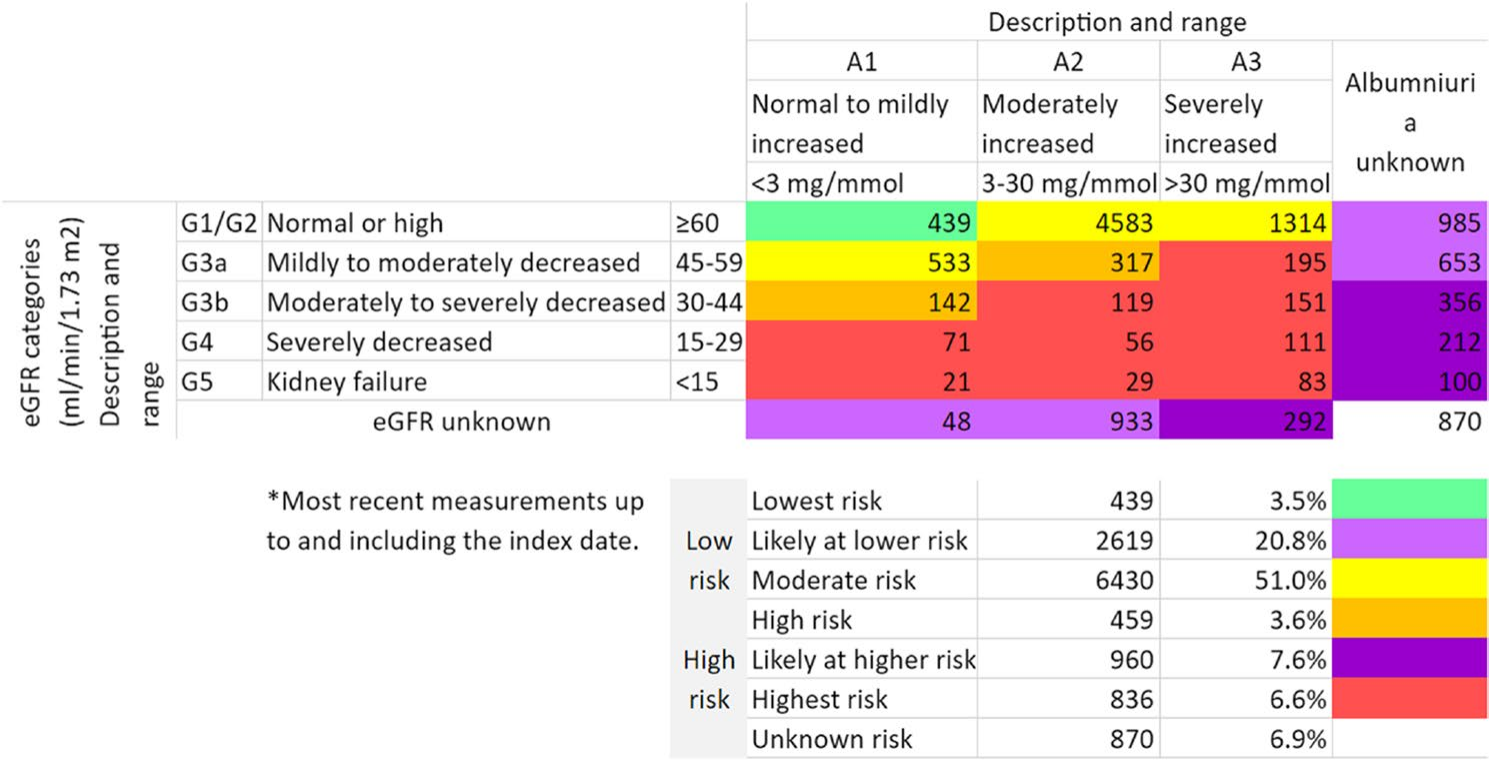
Placement on CKD heat map at baseline

The mean Elixhauser Comorbidity Index was 6.7 ± 3.3. Most individuals (75.2%) in the cohort were considered low or moderate risk of kidney failure at baseline (Fig. 1). In addition, Overall, 35.5% of those diagnosed were at unknown risk because of missing eGFR or albuminuria results, which brings into question the quality of PHC. In addition, 18.1% of those diagnosed with CKD were considered at high risk of developing ESKD.

### Laboratory testing

FN individuals with CKD who reside in communities served by Nursing Stations when supplemented by OHS or DIP consistently had the highest crude and adjusted rates of laboratory testing (Table 2).

**Table 2:** Per person-year crude and adjusted* rates of laboratory testing by community of residence

	Serum Creatinine		Urine ACR		Serum Potassium		HgbA1C		Cholesterol		Serum Albumin	
	CR	AR	CR	AR	CR	AR	CR	AR	CR	AR	CR	AR
Health Centre + DIP	1.81	1.92	0.95	0.91	1.64	1.74	1.09	1.10	0.69	0.69	0.98	1.02
Health Centre Only	2.05	1.98	0.86	0.83	1.88	1.80	1.17	1.14	0.72	0.70	1.07	1.01
Health Office + DIP	1.99	2.07	0.93	0.91	1.90	1.96	1.15	1.22	0.74	0.78	1.04	1.07
Health Office + OHS	1.66	1.96	0.67	0.69	1.52	1.79	0.88	0.98	0.48	0.52	0.77	0.89
Health Office Only	1.78	1.78	0.76	0.77	1.69	1.67	0.97	1.00	0.57	0.58	0.93	0.91
Nursing Station + DIP	2.30	2.40	1.03	0.99	2.20	2.28	1.29	1.32	0.76	0.77	1.42	1.44
Nursing Station + DIP + OHS	2.24	2.65	1.04	1.07	2.06	2.44	0.95	1.06	0.65	0.72	1.34	1.53
Nursing Station + OHS	2.87	3.06	1.17	1.01	2.73	2.94	1.60	1.49	1.01	0.93	1.50	1.59
Nursing Station Only	1.80	1.88	1.00	0.93	1.71	1.78	1.45	1.39	0.83	0.80	1.15	1.19
Off Reserve Rural	1.98	2.03	0.83	0.84	1.86	1.90	1.08	1.10	0.58	0.60	1.03	1.04
Off Reserve Urban	2.40	2.29	0.83	0.81	2.24	2.13	0.67	0.67	0.35	0.36	1.08	1.00

Negative binomial regression models adjusted for age, sex, comorbidities, and baseline kidney function showed they were consistently more likely to receive follow-up serum creatinine (OR 1.37, 95% CI: 1.30–1.45, *p* < 0.001), urine ACR (OR 1.22, 95% CI: 1.16–1.28, *p* < 0.001), serum potassium (OR 1.40, 95% CI: 1.32–1.49, *p* < 0.001) testing than individuals who lived in communities served by Nursing Stations alone, Health Centres, Health Offices, or Off-Reserve (Table 3).

**Table 3:** Comparing nursing stations + OHS and/or DIP versus all other communities for rates of laboratory testing adjusted for age, sex, eGFR, urine ACR, and elixhauser comorbidity index

	OR (95% CI)	*p*-value
Serum Creatinine	1.37 (1.30–1.45)	< 0.001
Urine ACR	1.22 (1.16–1.28)	< 0.001
Serum Potassium	1.40 (1.32–1.49)	< 0.001
HgbA1C	1.48 (1.42–1.55)	< 0.001
Cholesterol	1.57 (1.49–1.64)	< 0.001
Serum Albumin	1.53 (1.42–1.65)	< 0.001

### Nephrology consults

FN individuals with CKD who reside in communities served by Nursing Stations supplemented by OHS or DIP consistently had the highest crude and adjusted rates of nephrology consults (Table 3). Negative binomial regression models adjusted for age, sex, comorbidities, and baseline kidney function showed they were consistently more likely to visit a nephrologist (OR 1.77, 95% CI: 1.30–2.40, *p* < 0.001) than individuals who lived in communities served by Nursing Stations alone, Health Centres, Health Offices, or Off Reserve (Table 5).

### Primary healthcare

FN individuals with CKD who reside in communities served by Nursing Stations supplemented by OHS or DIP, consistently had the lowest crude and adjusted rates of PHC visits (Table 3). Negative binomial regression models adjusted for age, sex, comorbidities, and baseline kidney function showed they were consistently less likely to visit a PHC practitioner (OR 0.55, 95% CI: 0.52–0.57, *p* < 0.001) than individuals who lived in communities served by Nursing Stations alone, Health Centres, Health Offices, or Off Reserve (Table 5). This finding may be misleading, since PHC provided by nurses in Nursing Stations is not included in administrative data.

FN individuals with CKD who reside in communities served by Health Offices alone were less likely to visit a PHC practitioner (OR 0.83, 95% CI: 0.75–0.91, *p* < 0.001) than individuals who lived in communities served by Health Offices supplemented by DIP. Similarly, FN individuals with CKD who reside in communities served by Health Centres alone were less likely to visit a PHC practitioner (OR 0.88, 95% CI: 0.80–0.97, *p* = 0.010) than individuals who lived in communities served by Health Centres supplemented by DIP.

### Hospitalizations

FN individuals with CKD who reside in communities served by Nursing Stations, when supplemented by OHS or DIP, consistently had the lowest crude rates, but higher adjusted rates, of hospital admissions (Table 4). Negative binomial regression models adjusted for age, sex, comorbidities, and baseline kidney function showed they were consistently more likely to receive hospital care (OR 1.34, 95% CI: 1.20–1.49, *p* < 0.001) than individuals who lived in communities served by Nursing Stations alone, Health Centres, Health Offices, or Off Reserve (Table 5). There was no statistical difference between the two groups for ACSC hospitalizations.

**Table 4:** Per person-year crude (CR) and adjusted* rates (AR) of nephrology consults, primary healthcare visits, and hospitalizations by community of residence

	Nephrology Consults		Primary Care		Hospitalization		ACSC Hospitalization		Hospital 30-day Readmission	
	CR	AR	CR	AR	CR	AR	CR	AR	CR	AR
Nursing Station Only	0.29	0.22	11.63	11.07	0.62	0.58	0.43	0.37	0.18	0.16
Nursing Station + DIP	0.41	0.29	7.40	7.16	0.64	0.58	0.38	0.32	0.17	0.14
Nursing Station + DIP + OHS	0.56	0.44	6.10	6.96	0.49	0.54	0.33	0.38	0.11	0.12
Nursing Station + OHS	0.39	0.33	4.70	5.88	0.47	0.62	0.27	0.36	0.11	0.15
Health Centre Only	0.38	0.28	13.31	12.18	0.49	0.42	0.45	0.36	0.11	0.08
Health Centre + DIP	0.46	0.35	11.56	11.29	0.60	0.57	0.57	0.50	0.14	0.13
Health Office Only	0.39	0.26	14.90	13.31	0.60	0.50	0.52	0.40	0.15	0.11
Health Office + DIP	0.39	0.30	16.20	14.19	0.77	0.63	0.71	0.54	0.19	0.14
Health Office + OHS	0.14	0.10	5.12	5.52	0.59	0.61	0.42	0.43	0.19	0.18
Off Reserve Rural	0.27	0.20	11.73	10.62	0.50	0.43	0.42	0.33	0.11	0.08
Off Reserve Urban	0.35	0.25	13.40	12.53	0.47	0.40	0.36	0.30	0.10	0.07

*Abbreviations*: *ACR* albumin to creatinine ratio, *AR* adjusted rates, *CR* crude rates

*Adjusted rates were adjusted for age, sex, Elixhauser comorbidity index, and CKD severity in Poisson regression models

**Table 5:** Comparing nursing stations + OHS and/or DIP versus all other communities for rates of nephrology consults, primary care visits, and hospitalizations adjusted for age, sex, eGFR, urine ACR, and elixhauser comorbidity index

	OR (95% CI)	*p*-value
Nephrology Consults	1.77 (1.30–2.40)	< 0.001
Primary Care Visits	0.55 (0.52–0.57)	< 0.001
Hospitalizations	1.34 (1.20–1.49)	< 0.001
ACSC Hospitalizations	1.09 (0.94–1.26)	0.27

As with PHC access, FN individuals with CKD who reside in communities served by Health Offices alone were less likely to be admitted to the hospital (OR 0.79, 95% CI: 0.63–0.99, *p* = 0.039) than individuals who lived in communities served by Health Offices supplemented by DIP. Similarly, FN individuals with CKD who reside in communities served by Health Centres alone were less likely to be admitted to hospital (OR 0.69, 95% CI: 0.55–0.87, *p* = 0.002) than individuals who lived in communities served by Health Centres supplemented by DIP. FN individuals with CKD living in communities served by Health Centres alone were also less likely to access hospital care for an ACSC versus those who reside in communities supplemented by DIP (OR 0.65, 95% CI: 0.47–0.90, *p* = 0.009).

### Prescription drugs

Overall, there was an increase in prescriptions of ACE-inhibitors/ARBs (58.0% vs. 62.37% *p* < 0.001), statins (40.4% vs. 44.9% *p* < 0.001), and other anti-hypertensives (43.2% vs. 48.0%) between the pre-index and post-index period (Table 6). Health Centre Only, Health Centre + DIP, Nursing Station + OHS, Nursing Station Only, and those Off Reserve also saw across the board statistically significant increase in those prescriptions between the pre-index and post-index period.

**Table 6:** Comparing nursing stations + OHS and/or DIP versus all other communities for prescription drug utilization

Prescription	ACEi/ARB			Statin			Other anti-hypertensive		
	% Pre	% Post	*p*-value	% Pre	% Post	*p*-value	% Pre	% Post	*p*-value
Overall	57.95%	62.37%	< 0.001	40.36%	44.91%	< 0.001	43.18%	48.01%	< 0.001
Nursing station only	73.08%	76.11%	< 0.001	54.51%	56.46%	0.026	52.59%	57.87%	< 0.001
Nursing station + DIP	66.90%	68.32%	0.33	42.72%	46.68%	0.001	46.11%	52.62%	< 0.001
Nursing station + DIP + OHS	43.56%	49.50%	0.109	36.63%	41.58%	0.059	41.58%	40.59%	0.8
Nursing Station + OHS	55.07%	65.34%	< 0.001	32.62%	41.74%	< 0.001	33.41%	39.29%	< 0.001
Health Centre only	62.87%	67.51%	< 0.001	40.36%	44.91%	< 0.001	49.58%	53.16%	< 0.001
Health Centre + DIP	60.33%	66.50%	< 0.001	43.67%	48.17%	0.002	46.83%	54.50%	< 0.001
Health Office + DIP	60.42%	62.20%	0.21	46.69%	49.92%	0.015	48.14%	53.31%	< 0.001
Health Office + OHS	44.32%	47.16%	0.3	35.23%	37.50%	0.157	30.68%	40.34%	< 0.001
Health Office Only	66.02%	65.05%	0.42	43.69%	46.97%	0.005	50.36%	52.91%	0.06
Off Reserve Rural	60.65%	62.97%	0.013	44.84%	48.26%	< 0.001	49.81%	52.32%	0.007
Off Reserve Urban	47.14%	51.50%	< 0.001	31.91%	36.27%	< 0.001	38.07%	42.03%	< 0.001

## Discussion

Remoteness is not a risk factor for FN in Canada, when access to PHC is primarily determined by FN-managed health services. These services tend to be more proactive in screening and follow-up of patients, resulting in better outcomes. It is also possible that FNs seek care more readily when services are provided by FN and allied providers. Addressing the rise in premature mortality experienced by FN from kidney diseases require greater investments in FN-centric PHC, that is locally managed. Our findings are consistent with previous research focused on on-reserve health services and outcomes [17, 19–22, 46, 72].

This study has many strengths: population-level administrative data allows the study of the entire registered First Nations population in the province rather than a sample that can introduce bias. This allowed us to run multiple GEE models and conduct separate analyzes of acute, chronic and ACSCs and identify differences in distinct communities served by different models of PHC. Our retrospective design also allowed for a longer study period and there was minimal loss to follow-up. Our study also has a number of limitations. First, administrative data do not provide information on factors such as quality of care in the community, delays in diagnosis, and transportation problems in the community that likely influence ACSC hospitalization. We are also unable to assess whether patients who were prescribed medication and had scheduled follow-up appointments might have opted/being precluded from adhering to treatment plans. Still, there is no reason to assume that there is a systematic bias in the distribution of these factors among PHC models. Further, in this patient-level analysis, patients were assigned to the community in which they lived at the time of diagnosis. Because the lifetime care trajectory of individuals is heavily influenced by access to First Nations led services in their home community such as DIP and OHS, and because we did not exclude health services received outside community from our count of outcomes, we used an intention-to-treat approach for exposure to community of residence and did not censor when an individual changed addresses. Even community members who move often travel back and forth to their home community and are still eligible to receive services there. During the study period, only 12% of individuals in the study had registered an address change and of those, approximately 33% moved back to their home community. As a result, censoring for changes in address did not meaningfully affect the analysis.

Additionally, there were limitations to the laboratory data. We did not have access to laboratory tests conducted in any private laboratories in Manitoba and due to due to missing laboratory data at baseline, we had to analyze eGFR and urine ACR as categorical variables rather than continuous variables in our models. However, Shared Health Diagnostic Services is estimated to conduct > 70% of laboratory tests province-wide, including all urine ACR testing. Further, including patients with missing laboratory data allowed us include all patients who met the inclusion criteria in the analysis and only 6.9% of patients were considered to be at unknown risk on the CKD heat map. Finally, PHC visits may have been undercounted in communities served by Nursing Stations as some of those services may have been billed to the Federal Government and thus not captured in provincial administrative data.

As mentioned, current Clinical Practice Guidelines (CPG) created by the Manitoba Renal Program (MRP), recommend yearly screening for all at risk individuals, including all FN individuals over 10 years of age [57]. Services provide in Nursing Stations and off-reserve, reflect a baseline of services provided to a majority of FNs living with CKD. Our results clearly show that this level of care is insufficient to meet CPG requirements.

There are a number of upstream initiatives currently in place to supplement existing PHC, such as the federally-funded DIP mentioned above. Beyond increased screening, the DIP is working to overcome barriers to access to a comprehensive, coordinated and integrated diabetes care and treatment service for limb, eye, cardiovascular and kidney complications.

While these initiatives are invaluable and are improving outcomes, PHC delivered off-reserve will continue to play an important role in meeting FN individuals’ health care needs. Our findings suggest that the better resourced part of the PHC system, those provided by FPs practicing off-reserve, funded through a fee-for-serve model and not capped, underperforms the on-reserve healthcare system when supplemented by OHS and DIP. This further suggests that the on-reserve healthcare system, as designed, staffed and funded, is insufficiently resourced to meet needs. This finding is consistent with previous studies [17–19, 21, 22, 51]. An implication of our findings is that off-reserve PHC providers need to step up in ensuring that kidney-proactive modalities are fully extended to FNs. This may require additional investments and some creativity given current workforce shortage.

Further, and to locate our findings within the broader context of how we decided to frame this paper, we remain concerned with current trends in rates of CKD in Manitoba FN communities, and with current services inability to turn the tide. Beyond PHC, investment in addressing systemic racism remains essential. All Canadian citizen should have equitable access to the determinants of health. For FN peoples, policies and priorities perpetuating systemic racism also perpetuate ill health, including early diagnoses of CKD. While essential, PHD is only one determinants of health.

### Conclusions and recommendations

This study reports that FN patients with CKD experience more responsive care when this care is delivered by either FN-governed health services (DIP), or Indigenous and non-Indigenous providers working for OHS. These programs and services have historically been under-resourced to meet ever expanding needs [73, 74]. PHC provided off-reserve, which is primarily delivered by non-Indigenous providers with little accountability for outcomes, seems to be underperforming in comparison to DIP and OHS with regards to kidney health for FN: the best resourced systems seem to be performing worse.

We conclude that improvements at four different levels are required to improve FN kidney health outcomes. *First*, communities with better access to PHC provided by First Nation-centric providers, experience better renal care follow up: this is independent of remoteness. The implication is that expanding such system, in terms of resources, will have a positive impact on kidney health outcomes. *Second*, off-reserve PHC is and will remain an important source of care for FNs for years to come: off-reserve PHC providers should be held accountable for the quality of care or lack of quality of care provided. *Third*, improving screening and treatment pathways is key: this requires addressing systemic and interpersonal racism in the health care system, improving access to screening and follow up. *Finally*, beyond PHC, addressing Indigenous determinants of health is key. This requires investments in safe housing and water, and addressing interpersonal and system racism at all levels.

Kidney disease is preventable, and decline can be delayed or halted with proper PHC. To date, Manitoba has had a dismal record of impact on renal disease progression for First Nations. We conclude with the following recommendations:

1. PHC provided outside of First Nation communities (better resourced than services provided in First Nation communities) is performing worse. Improving First Nations health outcomes will require increased accountability of off-reserve PHC providers.
2. Communities with better access to PHC provided by First Nation-sensitive providers, experience better renal care follow up: this is independent of remoteness. Expanding and resourcing such systems will have a positive impact on kidney health outcomes.
3. Clinical data is being collected across multiple jurisdictions (federal, provincial, FN)that cannot be accessed by all providers (lab data, blood pressures, care provided on-reserve, etc.), undermining quality and continuity of care. A province wide electronic health system tracking labs and basic medical data is imperative to bridge this gap.
4. PHC providers and the system that supports them must be educated, resourced and held accountable to implement FN-centric clinical practice guidelines with regards to screening and follow-up.
5. Patients have the right to access and understand their own health data, be empowered in their own decision-making and improve the care they receive from health providers [75]. A virtual platform needs to be created to allow all Manitobans to access their medical chart and laboratory results, and education needs to be provided to enable patients to understand the information they are accessing.
6. Current referrals systems are insufficient. A mechanism of automatic patient referrals where results suggest renal disease requiring specialist attention should be developed to better support PHC providers.
7. Current renal outcomes are in part associated with a lack of cultural safety. Addressing systemic and interpersonal racism in the health care system is key. All actions should align with Manitoba’s Indigenous health quality framework [76].
8. Beyond PHC, addressing determinants of health (housing, poverty, language, self-determination for examples) is key [77].

It is important to note that better primary health care, as provided by FNHSSM and the DIP, can mitigate the impact of systemic racism, to some extent. Systemic racism remains the most significant determinant of health perpetuating inequities. Ending systemic racism require systems’ change, and depend on federal and provincial governments’ goodwill.

## Data Availability

No datasets were generated or analysed during the current study.

## Abbreviations

ACSC: Ambulatory care sensitive conditions
ACE: Angiotensin-converting-enzyme inhibitors
CKD: Chronic kidney disease
CPG: Clinical Practice Guidelines
DIP: Diabetes Integration Project
ESKD: End stage kidney disease
eGFR: Estimated glomerular filtration rate
FPs: Family physicians
FN: First Nation
FNHSSM: First Nations Health and Social Secretariat of Manitoba
HIPC: Health Information Privacy Committee
HIRGC: Health Information Research Governance Committee
HgbA1c: Hemoglobin a1c
MRP: Manitoba Renal Program
OR: Odds ratio
OHS: Ongomiizwin Health Services
PHC: Primary healthcare
SES: Socio-economic status
ACR: Urine albumin to creatinine ratio
